# High-Resolution Anorectal Manometry as a Screening Tool for Hirschsprung’s Disease: A Comprehensive Retrospective Analysis

**DOI:** 10.3390/jcm13051268

**Published:** 2024-02-23

**Authors:** Oliver Sowulewski, Magdalena Bubińska, Agnieszka Zagierska, Maciej Zagierski, Agnieszka Szlagatys-Sidorkiewicz

**Affiliations:** Department of Paediatrics, Gastroenterology, Allergology & Paediatric Nutrition, Medical University of Gdańsk, 80-210 Gdańsk, Poland; hrynekmagda@gumed.edu.pl (M.B.); agnieszka.zagierska@gumed.edu.pl (A.Z.); maciej.zagierski@gumed.edu.pl (M.Z.); agnieszka.szlagatys-sidorkiewicz@gumed.edu.pl (A.S.-S.)

**Keywords:** Hirschsprung’s disease, high-resolution anorectal manometry, contrast enema, aganglionosis, HR-ARM, ARM, HD

## Abstract

Hirschsprung’s disease (HD) is characterized by a congenital absence of enteric ganglion cells in the intestine, posing challenges in diagnosis, particularly in pediatric patients. The gold standard, rectal suction biopsy (RSB), carries risks, prompting an exploration of non-invasive alternatives such as high-resolution anorectal manometry (HR-ARM) for HD screening. We conducted a retrospective analysis of 136 patients suspected of HD between 2018 and 2022, which were stratified into three age groups: ≤12 months, ≤24 months, and >24 months. Criteria for suspicion included delayed meconium passage, unresponsive chronic constipation, and abnormal prior test results. HR-ARM, supplemented by additional tests, confirmed 16 HD cases. HR-ARM exhibited 93.75% sensitivity, 89.47% specificity, 99.03% negative predictive value (NPV), and 55.56% positive predictive value (PPV). Notably, HR-ARM consistently performed well in patients ≤ 2 years old but demonstrated reduced efficacy in older children, which was likely due to complications from chronic constipation. This study underscores HR-ARM’s promise as a non-invasive HD screening tool, especially in younger patients. However, its limitations in older children warrant consideration. Establishing standardized protocols, particularly for assessing the recto-anal inhibitory reflex, is crucial. Further research is imperative to optimize HR-ARM’s diagnostic role across varied age groups in HD assessment.

## 1. Introduction

Hirschsprung’s disease (HD) is a congenital developmental disorder characterized by the absence of enteric ganglion cells in submucosal and myenteric plexuses of the intestine [1]. The direct cause of HD is improper migration of neural crest cells to the intestinal wall during the fetal development. The majority of cases (more than 80%) affect the distal part of large bowel, primarily the rectum and sigmoid colon [2]. It results in the intestinal contraction and can be one of the causes of constipation. The estimated prevalence of HD is 1.09–2 per 10,000 live births with a male predominance and a sex ratio of approximately 4:1 [3,4].

Symptoms of HD typically manifest in the neonatal period, and the diagnosis is established within the first three months of life in more than 90% of cases. However, in some instances, symptoms may appear later [5]. The typical clinical presentation of Hirschsprung’s disease includes delayed meconium passage, severe constipation without improvement after treatment, abdominal distention, vomiting and failure to thrive. Fecal stasis can lead to bacterial overgrowth and severe complications such as Hirschsprung-Associated Enterocolitis (HAEC), which is also the leading cause of death in this patient group [6].

Therefore, it is crucial to promptly diagnose HD and initiate treatment, which means surgical intervention [7]. The gold standard in the diagnostic process of HD is histological evaluation of the specimens obtained during rectal suction biopsy (RSB) with appropriate staining. The absence of ganglia in submucosa confirms the diagnosis, but this requires an experienced pathologist, while HD is a relatively rare disease [8]. Nowadays, RSB can be performed without general anesthesia, but it also depends on the clinical experience of specific clinicians [9]. In some cases, RSB may give inconclusive results due to inadequate material, necessitating the repetition of RSB or the performance of a full-thickness biopsy (FTB). RSB, like any surgical procedure, carries the risk of complications such as bleeding, perforation or infection, particularly in newborns and infants. RSB-related adverse events are rare (0.65%), but they can be severe [10]. Therefore, patient selection for RSB must be carefully considered. Additional tests such as contrast enema (CE) and anorectal manometry aid in determining which patients should undergo RSB [11]. CE may suggest HD based on the transition zone between the ganglionic, contracted segment of the intestine and the dilated segment above [12,13]. However, CE is associated with radiation exposure to the sensitive urogenital area and may not reveal the transition zone in ultra-short Hirschsprung’s disease or in early HD without colonic dilatation [14]. Anorectal manometry involves the insertion of a catheter through the anus to assess the presence of the rectoanal inhibitory reflex (RAIR) in response to rectal distention [15]. In HD, characterized by aganglionosis, the absence of RAIR may be indicative for the diagnosis [16]. The newest tool, high-resolution anorectal manometry (HR-ARM) with an increased number of pressure sensors, appears to be more specific than conventional anorectal manometry and is gaining wider use [17]. HR-ARM is a relatively safe and non-invasive test [18]. However, the role of anorectal manometry in the diagnostic process of HD remains debated, and data are limited.

Clear guidelines for the qualification of patients for RSB are still missing. Therefore, we conducted a retrospective evaluation of a group of patients suspected of having HD, who underwent CE and HR-ARM examinations. The aim of our study was to assess whether HR-ARM is a sufficient screening tool for qualifying patients suspected of having HD for RSB. 

## 2. Materials and Methods

### 2.1. Subjects

This was a retrospective cohort study that enrolled patients with clinical suspicion of HD who underwent high-resolution anorectal manometry (HR-ARM) in the Department of Paediatrics, Gastroenterology, Allergology & Paediatric Nutrition in Medical University of Gdansk between 2018 and 2022. The criteria for HD suspicion included delayed meconium passage, onset of constipation during infancy, chronic constipation (CC) unresponsive to conservative treatment, a history of enterocolitis or abnormal results from prior examinations such as contrast enema, ultrasound and plain X-ray. All patients suspected of having HD underwent HR-ARM; no individuals were definitively excluded from this examination. For those initially requiring alternative interventions, such as bowel washout or treatment for enterocolitis, HR-ARM was deferred until they achieved a stable clinical condition. In 3 patients, HR-ARM was nondiagnostic; those patients were excluded from calculations. 

### 2.2. HR-ARM Technique

**Figure 1:**
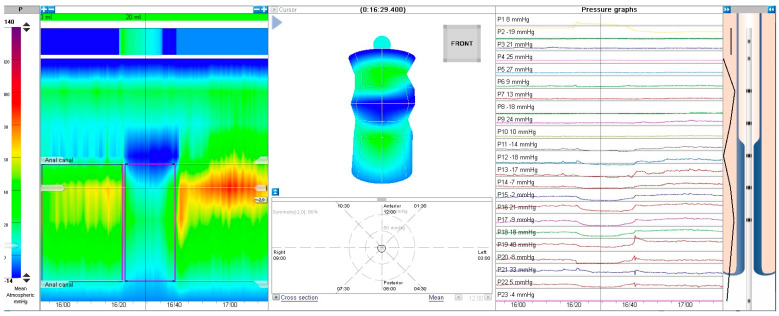
Recto-anal inhibitory reflex, HR-ARM.

**Figure 2:**
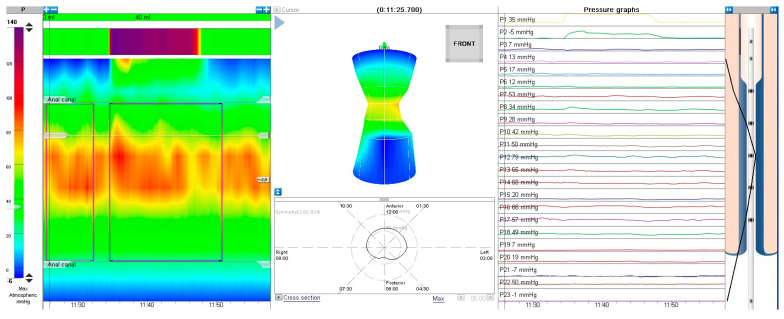
Absence of recto-anal inhibitory reflex, HR-ARM.

HR-ARM procedure was conducted using MMS Laborie equipment with a 24-channel solid-state catheter. The catheter was inserted to the appropriate depth as recommended by the manufacturer, and a water-based lubricant (OptiLube™) was used. The test was conducted on awake patients, and bowel preparation was not performed routinely unless the patient’s rectum was impacted with stool—in such cases, rectal enema was performed either the day before or a few hours prior to the examination. Each examination was performed by a single physician trained for HR-ARM. For most cases, we ran (if possible) a 3-minute adaptation period prior to the examination. The test involved assessment of anal canal resting pressure for ca. 30 s (this part was omitted in non-cooperating patients). Next, the presence of rectoanal inhibitory reflex (RAIR) was assessed by inflating the balloon with air volume starting with 5 mL and increasing by another 5 mL each time until RAIR was elicited, the patient presented discomfort or up to 60 mL. RAIR was considered present when a pressure drop of at least 25% or 10 mmHg was observed. Examples of present and absent RAIR are presented in Figure 1 and Figure 2.

### 2.3. Contrast Enema Technique

Contrast enema (CE) was conducted without prior bowel preparation. Diluted barium was infused rectally by a radiologist and X-ray images were captured during infusion and subsequently 24 h later. Indicators of HD included distal colon dilation, proximal narrowing or an abnormal recto-sigmoid ratio (>1). 

### 2.4. Diagnostic Pathway and Confirmation of Hirschsprung’s Disease

**Figure 3:**
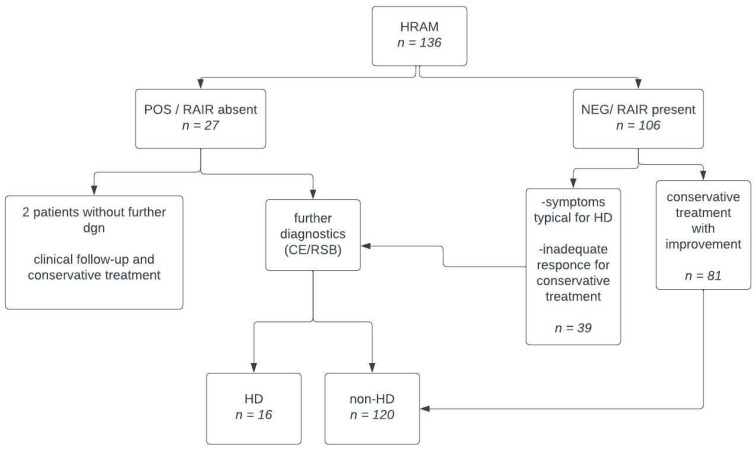
Diagnostic plan flowchart.

All patients with clinical suspicion of HD were examined with HR-ARM. For patients with absence of RAIR (positive test for HD), additional tests such as contrast enema (CE) and/or suction rectal biopsy (RSB) were performed. In cases when RSB was inconclusive, full-thickness biopsy (FTB) was carried out. In instances where RAIR was present, further diagnostics were not routinely pursued, and the patient was diagnosed as non-HD. However, exceptions were made for patients displaying typical HD symptoms (e.g., bloating, severe constipation, poor weight gain, progressive cachexia or enterocolitis). Such patients were referred for supplementary tests. Ultimately, the final confirmation of HD relied on the results of either rectal suction biopsy (RSB) or full-thickness biopsy (FTB). Conversely, the exclusion of HD was established through clinical follow-up or negative biopsy outcomes (Figure 3).

A secondary objective of our study was to conduct a comparative analysis of symptom prevalence between patients diagnosed with Hirschsprung’s disease (HD) and those without. Additionally, we compared HR-ARM metrics, such as anal canal length and mean resting pressure, across these patient groups.

### 2.5. Data Analysis and Statistical Methods

Analyses were performed for HR-ARM and CE for all patients and for subgroups stratified by age (≤12 months, ≤24 months, >24 months). Descriptive statistics were performed with use of medians, means, standard deviations and IQRs (interquartile ranges). The sensitivity, specificity, positive predictive value (PPV), and negative predictive value (NPV) along with 95% confidence intervals were calculated for HR-ARM and CE. Rates were compared with Fisher’s exact test. Means were compared with *t*-tests. All tests were two-sided. *p*-value of <0.05 was considered statistically significant. All analyses were performed with GraphPad Prism 10.0 (GraphPad Software LLC, Boston, MA, USA).

## 3. Results

During the study period, 136 subjects fulfilled the inclusion criteria and were analyzed. There were 55 females, and 81 were males. The age range of the patients was 6 days to 16 years old with a median age of 1 year (364 days). The majority of patients (89) were under the age of 2 years. Group characteristics are described in Table 1.

The most prevalent findings observed in children undergoing HR-ARM due to suspicions of HD (Table 2) were constipation −89.7% (116 out of 136), delayed meconium passage −14.7% (20 out of 136), abdominal distention −11% (15 out of 136), and a history of enterocolitis 0.44% (6 out of 136).

**Figure 4:**
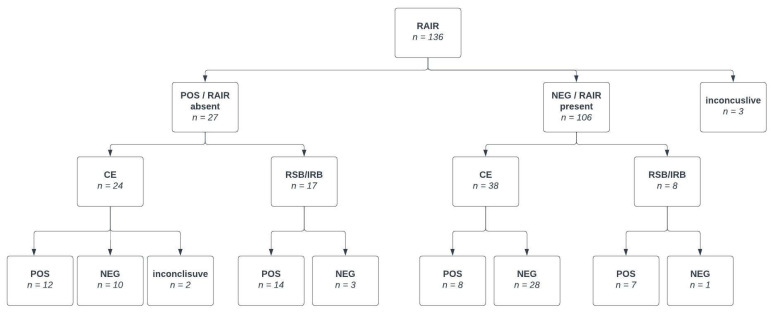
Flowchart presenting post HR-ARM pathway.

In this study, among the 136 patients who underwent HR-ARM, 27 individuals exhibited an absent RAIR (positive test for HD) (group A), while 106 patients displayed a present RAIR (negative HD test) (group B). Two patients had non-diagnostic RAIR results due to artifacts, and one test was considered inconclusive (Figure 4).

### 3.1. Group A—HR-ARM Positive for HD (Absence of RAIR)

Among the patients, 27 were identified as positive for HD screening through HR-ARM (no RAIR). For patients where the absence of RAIR was believed to be due to reasons other than HD (megarectum as a result of chronic constipation), RSB was not conducted—in these patients, a watch-and-wait approach was chosen, and subsequent clinical follow-up indicated no HD (the average age of these patients was 48.9 months). One patient died before the diagnostic process was completed, and a post-mortem examination confirmed aganglionosis. 

Out of the 27 patients initially suspected to have HD following HR-ARM, 16 were indeed diagnosed with HD, while the remaining 11 were confirmed to be healthy. Within this group, CE yielded a positive result for HD in 12 patients, of which two were false positives. Conversely, CE was negative for HD in 10 patients, three of whom were later diagnosed with HD. Two tests were inconclusive. 

### 3.2. Group B—HR-ARM Negative for HD (RAIR Present)

Of the 106 subjects who were tested negative for HD through HR-ARM (RAIR present), 38 patients underwent contrast enema and 8 underwent RSB. Notably, 105 anorectal manometries were true negatives. Hirschsprung’s disease was diagnosed in a single case despite the presence of RAIR; this anomaly was most likely attributed to technical errors during anorectal manometry. 

Among the 38 patients who underwent contrast enema, seven individuals received a positive HD result with only one being genuinely positive. CE yielded negative HD results in 26 patients, all of which were true negatives. 

CE and HR-ARM produced concordant outcomes in 10 cases, while HR-ARM consistently outperformed CE in cases of disagreement. Importantly, no patients exhibited a combined false negative result in both tests. There were no patients in whom both tests showed false negative result when combined (either one was positive). 

### 3.3. HRAM vs. CE 

#### 3.3.1. HR-ARM Performance

HR-ARM demonstrated true positivity in 15 cases, false positivity in 12, true negativity in 105 and false negativity in 1 case. The sensitivity of HR-ARM was 93.75%, and specificity was 89.47% with a negative predictive value (NPV) of 99.03% and a positive predictive value (PPV) of 55.56% (Table 2). Disease prevalence in this group was 12.03%.

#### 3.3.2. CE Performance

CE displayed true positivity 11 times, false positivity 9 times, true negativity 37 times, and false negativity 3 times. CE’s sensitivity stood at 78.57%, specificity at 80.04%, NPV at 92.5%, and PPV at 55% (Table 2). Disease prevalence in this group was 11.05%. 

**Figure 5:**
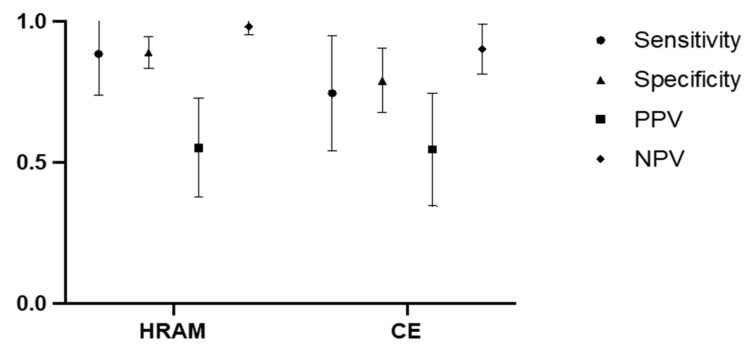
The 95%CI visual presentation of HR-ARM and CE performance in total population.

In the overall examined population, both HR-ARM and X-ray enema exhibited a substantial negative predictive value. HR-ARM consistently outperformed CE across all measured parameters (Figure 5).

### 3.4. Analysis of Patients in Different Age Groups

The results of HR-ARM and CE were further assessed by categorizing subjects into age groups: ≤12 months, ≤24 months, and >24 months. We have determined the sensitivity, specificity, PPV, and NPV in these groups (Table 3).

#### 3.4.1. Patients Aged ≤12 Months

Within this youngest group of 68 patients, all underwent HR-ARM, and 26 underwent CE. Among them, 12 patients were eventually diagnosed with HD. Both tests demonstrated nearly equal quality and utility with CE slightly edging out HR-ARM in NPV.

#### 3.4.2. Patients Aged ≤24 Months

This group comprised 89 subjects, all of whom underwent HR-ARM, and 37 of them received CE. Among this cohort, 15 out of 89 subjects were ultimately diagnosed with Hirschsprung’s disease. 

#### 3.4.3. Patients >24 Months Old

For patients aged over 2 years, 44 subjects underwent HR-ARM, and CE was performed in 23 out of 44. The diagnosis of HD was established in just 1 patient within this age group. Due to the limited number of HD cases diagnosed in patients over 2 years old, statistical analysis was not significant.

### 3.5. Results Summary 

In summary, regarding the utility of high-resolution anorectal manometry, our analysis has shown that the sensitivity and positive predictive value of HR-ARM were notably lower in children over 2 years of age. Consequently, the outcomes of the overall population analysis might be somewhat underestimated due to the inclusion of older patients within the analyzed group. We have summarized the gathered data for all age groups, encompassing both HR-ARM and contrast enema results in Table 3. 

**Figure 6:**
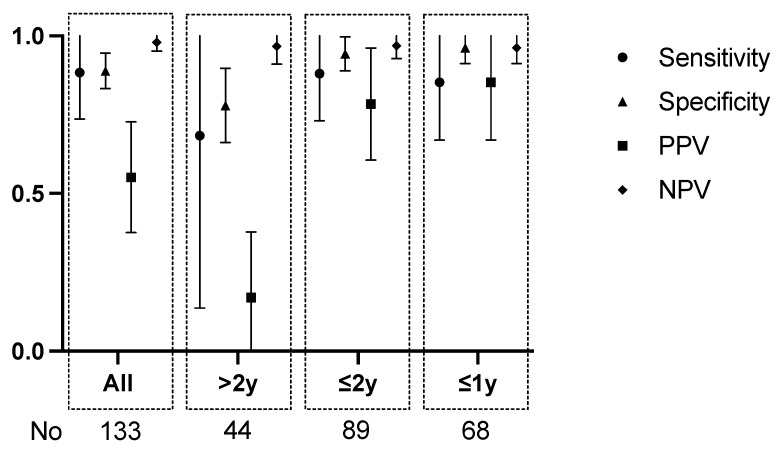
The 95% CI graphical presentation of sensitivity, specificity, PPV and NPV for HR-ARM in different age groups. (Detailed values of 95% CI can be found in Supplementary Material Table S3).

For patients aged ≤1 year and ≤2 years, there exists no significant disparity in the effectiveness of HR-ARM (Figure 6).

### 3.6. Additional Analysis

Symptom prevalence varied between patients with and without HD. Among the 116 patients with chronic constipation, 9.4% (11 patients) were ultimately diagnosed with HD. Among the 20 patients with delayed meconium passage, only 25% (5) were found to have HD. Abdominal distention was noted in 15 patients with 60% (9) of them being diagnosed with HD. Enterocolitis was reported in 6 cases, and 83.33% of those patients (5) were confirmed to have HD.

We also performed an analysis of the mean anal canal length and mean anal canal resting pressure measured during HR-ARM (Table 4). The average anal canal length measured 2.47 cm for patients with HD and 2.5 cm for healthy patients. Meanwhile, the mean anal canal resting pressure was 75.22 mmHg for individuals with HD and 65.61 mmHg for those without HD. However, our analysis did not reveal any statistically significant differences between these two groups.

## 4. Discussion

The majority of symptoms associated with HD emerge during infancy and can prompt significant concern among both parents and healthcare professionals. Symptoms such as constipation, vomiting and failure to thrive are non-specific, and diagnosis can be challenging. Most patients with HD experience delayed meconium passage (>24 h), but this is also not a specific symptom [19]. Premature neonates and some health individuals might have a history of delayed passage of meconium [3]. A late diagnosis of HD can result in enterocolitis and an extended hospital stay requiring antibiotic treatment. Moreover, the neonatal period is a challenging time for conducting invasive or numerous medical procedures due to the infant’s low weight and limited cooperation. Therefore, having a non-invasive screening tool appears to be an important goal in diagnostic process of HD, allowing for the exclusion of a serious underlying cause of the symptoms. 

For many years, contrast enema (CE) was recommended as the first diagnostic test for HD [20]. The authors report that its sensitivity and specificity in the diagnosis of HD vary 76–86.9% and 92.1–97%, respectively [21,22]. However, CE has limitations, as it involves irradiation of the urogenital area. Furthermore, in cases of early HD when there is no dilation yet, CE may not reveal any abnormalities [14]. In cases of ultra-short segment or total aganglionosis, diagnosis can also be challenging, although these represent a minority of all HD cases [22]. The test results are delayed due to the need to take the X-rays with the 24 h interval and an experienced radiologist assessment. 

Rectal suction biopsy stands as the gold standard in Hirschsprung’s disease diagnosis [8]. Presently, the procedure can be performed without general anesthesia as a bedside procedure [9]. However, not all medical centers adopt this practice. Frequently, RSBs are conducted under general anesthesia, including patients from our analysis. At present, it is recommended that each case undergo confirmation in biopsy. However, this method is not without its shortcomings. It is an invasive procedure carrying the risk of complications at a frequency of 0.65%, including serious ones such as bleeding at the sampling site requiring surgical intervention, and the complication rate is the highest in newborns and infants [7]. Moreover, the adequacy of the collected material for assessment may sometimes be insufficient, necessitating repetition up to 17–22% of procedures [23,24] or performance of a full-thickness biopsy, thus delaying diagnosis and requiring general anesthesia. Additionally, in ultra-short segment cases, capturing the precise affected bowel fragment can be challenging. In the presence of complications such as enterocolitis, the procedure may be difficult to conduct. Owing to concerns voiced by the referring physician about RSB-related complications and the potential for false-negative results, there exists a risk of underdiagnosing HD [25]. The specificity and sensitivity of RSB are notably high, 99.41% and 98.84%, respectively [10], and presently, there is no diagnostic alternative available. One study calculated a lower sensitivity of 81%, while specificity was similar at 97% [24]. However, an instrument that appears to meet the criteria for a screening tool and excluding HD is high-resolution anorectal manometry (HR-ARM). It offers a non-invasive, safe, and expeditious option. Results can be promptly assessed. 

The data describing HR-ARM’s ability to exclude or confirm HD are still limited, and large cohort studies are missing. 

In 2014, Tang et al. performed HR-ARM on 180 asymptomatic newborns and 16 newborns suspected of having HD. RSB was administered following positive tests to confirm or exclude HD. Their evaluation demonstrated a sensitivity of 89% and specificity of 83% with positive and negative predictive values of 89% and 83%, respectively. It is worth noting that their study primarily focused on newborns, and thus, these results are comparable to the ≤12 month subgroup of the current study [18]. 

In a 2005 study by Lorijn et al., the diagnostic accuracy of three tests (contrast enema, anorectal manometry, and rectal suction biopsy) for Hirschsprung’s disease in infants was compared. They conducted a prospective analysis involving 111 infants suspected of having HD. The results indicated that rectal suction biopsy exhibited the highest sensitivity (93%) and specificity (100%) for diagnosing HD while also having the lowest rate of inconclusive results. However, these values were not significantly different from those of anorectal manometry (sensitivity 83%, specificity 93%) [21]. In this study, the authors did not focus on the NPV or PPV of each test. The major drawback of this paper is using conventional manometry, which is nowadays outdated and inferior to HR-ARM.

In 2018, Meinds, Trzpis, and Broens conducted a comparative analysis of the diagnostic capabilities of conventional anorectal manometry (ARM) and rectal suction biopsy for Hirschsprung’s disease. Their prospective study included 105 patients who underwent manometry, followed by RSB, when HD was suspected. The study revealed that anorectal manometry exhibited a sensitivity equivalent to that of RSB at 97% with a specificity of 74%, a 100% negative predictive value, and a positive predictive value of 56%. Their conclusion supported the idea that ARM could serve as a viable screening tool for HD. However, it is noteworthy that their study lacked a standardized protocol. During the course of their research, they modified the test protocol, and it was found that altering the volume of the balloon significantly increased specificity. This suggests that the assessment of anorectal manometry as a tool for excluding HD depends on the established protocol. Furthermore, Meinds did not observe an age-related dependency on age in the NPV. This could be due to the fact that all cut-offs were <2 yo [16]. 

Our study, compared to available analyses, included a relatively large study group. All HR-ARM tests were performed in a unified protocol, which is similar to protocol published and recommended by BSPGHAN in 2020 [26] (our retrospective analysis involves patients between 2018 and 2022). Another difference is the equipment used to perform an examination. We used high-resolution anorectal manometry. It is new method working with more pressure channels and better effectiveness.

Our study indicates that HR-ARM is a good screening tool in the diagnostic process of Hirschsprung’s disease. We assessed its specificity and sensitivity in different age groups and proved that in patients < 2 years old, this method is viable to exclude HD. HR-ARM has a greater specificity especially in the group of children under two years of age. Specificity may be lower in children over two years of age due to complications of chronic constipation, such as rectal distension. In this case, the maximum volume of the balloon may not be sufficient. The sensitivity of HR-ARM is higher in younger patients, and we think that the low sensitivity presented for the total group is probably driven by the group of patients > 2 yo in whom the number of false positive results greatly increases. It may be caused by the fact that the maximum volume used to elicit RAIR was not enough to trigger rectum distension; thus, we recommend further studies to determine if eliciting RAIR with a higher volume is safe and efficient in children.

We agree that the gold standard in the diagnostic process of HD remains RSB. It is an invasive procedure, but at present, it is necessary to confirm the diagnosis. Friedmacher et al. in their systematic review showed that the overall complications rate of RSB is low (0.65%), but the greatest risk is in newborns and infants [10]. Our results indicate that HRAM as screening tool may reduce the number of complications in that age group. We suggest that RSB should be carried out for every patient < 2 yo with an absence of RAIR in HRAM. For older children, the decision should be made individually, taking into account the duration of symptoms and their severity. HRAM as a non-invasive method is easier and safer to perform than RSB. In most cases, it does not require any previous bowel’s preparation and may be realized during a one-day stay at the hospital or in the outpatient clinic. It may also reduce costs connected with hospitalization. 

HR-ARM is a safe and minimally invasive procedure, and complications associated have not yet been reported. This makes it a well-tolerated diagnostic tool, especially in pediatric patients. The availability of HR-ARM is increasing, but this test should be conducted in reference centers with experience diagnosing and treating HD. 

Our study has some limitations. The group of patients > 2 yo was relatively small, and the results in that group turned out to be statistically insignificant. That makes it unfeasible to compare two age groups. It is caused by the fact that almost 90% of the diagnoses are made in the neonatal period, so it is challenging to collect older patients. Furthermore, the diagnostic process was not uniform in all patients. (Results for patients who underwent both HR-ARM and CE can be found in Aupplementary Materials—Tables S1 and S2). This was due to the lack of clear guidelines regarding how to diagnose the Hirschsprung’s disease step by step. Additional tests were carried out in a different order, which could have influenced the interpretation of the particular results; however, each case of HD was confirmed in the biopsy. In addition, the retrospective nature of the study makes it difficult to collect complete data about patients and their symptoms. Therefore, the frequency of individual symptoms may differ from the actual one. However, our study focused on the evaluation of HR-ARM as a screening method and the possibility of replacing CE by HR-ARM. Finally, it is important to note that patients exhibiting negative RAIR in HR-ARM were not routinely referred for further diagnostics, such as CE or RSB. We are aware that this approach might raise concerns about the potential omission of cases that could have had positive findings in these additional tests and finally affect NPV. However, this is not probable, as those patients were under continuous observation and did not present typical symptoms further in the follow-up. 

In our study, we used a gradual increase in volume by 5 mL to trigger RAIR. This method aligns with the current recommendation from the British Society of Pediatric Gastroenterology, Hepatology, and Nutrition (BSPGHAN) [26]. For older children dealing with chronic constipation, it might be wise to consider using larger volumes, exceeding 60 mL, to trigger RAIR. In such cases, ongoing evaluation and further testing are necessary to make an accurate diagnosis.

Guidelines on which patients should definitely be referred for RSB are missing. Contrast enema and RSB are commonly used methods. In our research, we wanted to place high-resolution manometry in a possible diagnostic pathway for patients suspected of HD, especially those that are not presenting typical symptoms, and there are doubts regarding whether they should undergo RSB. Our findings, similar to those of Mendis et al. and Tang et al. [16,18], support the idea that high-resolution anorectal manometry can be a useful way to screen for Hirschsprung’s disease (HD). Its impressive negative predictive value (NPV) shows that it is a good first choice for diagnosing patients suspected of having HD. We recommend that in those patients, HR-ARM as a safe and well-tolerated examination should be a first-choice screening test. Further research is needed to establish this standardized approach. 

## 5. Conclusions

We have found that HR-ARM is a consistent, repeatable, and acceptable method for screening for HD and qualifying patients for further diagnostics like RSB. It demonstrates high sensitivity, specificity, and NPV. This study is inexpensive, easy, and safe with its availability dependent on having the necessary equipment and expertise. In this research, we have demonstrated the significant role of HR-ARM in the diagnostic process and its contribution as a primary screening test in patients suspected of HD.

## Figures and Tables

**Table 1:** Group characteristics.

	Age (Months)	Weight (kg)	Height (cm)	Z-Score Weight	z-Score Height	z-Score Weight for Length
Median	11.750	9.050	77.500	−0.070	0.090	−0.340
Mean	27.589	12.154	81.359	−0.301	0.058	−0.443
SD	39.395	12.675	26.593	1.419	1.871	1.291
Min	0.200	2.550	46.000	−5.930	−6.070	−3.100
Max	192.60	110.00	172.000	2.780	4.530	2.730

**Table 2:** HR-ARM and contrast enema contingency table—total population.

	HR-ARM		CE	
	HD	Non-HD	HD	Non-HD
POS	15	12	11	9
NEG	1	105	3	37

**Table 3:** HR-ARM and CE summarized performance in different age groups.

	HR-ARM Performance						
	n	Sensitivity	Specificity	PPV	NPV	Likelihood Ratio	*p*-Value
TOTAL	133	93.75%	89.74%	55.56%	99.06%	9.141	<0.0001
≤12 months	68	91.67%	98.21%	91.67%	98.21%	51.33	<0.0001
≤24 months	89	93.33%	95.77%	82.35%	98.55%	22.09	<0.0001
>24 months	44	100%	79.95%	10%	100%	4.889	0.2222 ns
	CE Performance						
	n	Sensitivity	Specificity	PPV	NPV	Likelihood Ratio	*p*-value
TOTAL	60	78.57%	80.43%	55%	92.50%	4.016	0.0001
≤12 months	26	100%	75%	71.43%	100%	4	0.0002
≤24 months	37	84.62%	80.95%	73.33%	89.47%	4.442	0.0003
>24 months	23	0	81.82%	0	94.74%	0	>0.99 ns

**Table 4:** Mean anal canal length and pressure in HD vs. non-HD patients.

	HD	Non-HD
Mean anal canal length (cm)	2.47	2.52
Mean anal canal pressure (mmHg)	75.22	65.61

## Data Availability

The raw data supporting the conclusions of this article will be made available by the authors on request.

## Supplementary Materials

The following supporting information can be downloaded at: https://www.mdpi.com/article/10.3390/jcm13051268/s1, Table S1: Contingency table for patients undergoing both HR-ARM and CE; Table S2: HR-ARM and CE performance in patients undergoing both HR-ARM and CE; Table S3: Below tables presenting 95% CI for both tests divided into age-groups.
