# Supplementary material for: High-Resolution Anorectal Manometry as a Screening Tool for Hirschsprung’s Disease: A Comprehensive Retrospective Analysis
Source: J Clin Med. 2024 Feb 23;13(5):1268. doi: 10.3390/jcm13051268 (PMC10932072; doi:10.3390/jcm13051268)
Supplement: Supplementary file 1 [file jcm-13-01268-s001.zip › jcm-2807323-supplementary conversion confirmed.pdf]

Supplementary Materials

Presenting additional results—Table S1 and Table S2—patient distribution and HR-ARM and CE performance in patient who underwent both tests. Below we present tables with 95% CI for each patient group—graphical presentation in main body of the manuscript

Table S1. Contingency table for patients undergoing both HR-ARM and CE.

	**HR-ARM**		**CE**	
	**HD**	**non-HD**	**HD**	**non-HD**
POS	13	9	11	9
NEG	1	37	3	37

Table S2. HR-ARM and CE performance in patients undergoing both HR-ARM and CE.

	**n**	**Sensitivity**	**Specificity**	**PPV**	**NPV**
HR-ARM	60	92.85%	80.43%	59.09%	97.36%
CE	60	78.57%	80.43%	55.0%	92.5%

Table S3. Below tables presenting 95% CI for both tests divided into age-groups.

**HR-ARM Total**			**CE Total**		
	**value**	**95% CI**		**value**	**95% CI**
**Sensitivity**	0.9375	0.7167 to 0.9968	**Sensitivity**	0.7857	0.5241 to 0.9243
**Specificity**	0.8974	0.8293 to 0.9404	**Specificity**	0.8043	0.6683 to 0.8935
**PPV**	0.5556	0.3731 to 0.7241	**PPV**	0.55	0.3421 to 0.7418
**NPV**	0.9906	0.9485 to 0.9995	**NPV**	0.925	0.8014 to 0.9742
**HR-ARM ≤12 months**			**CE ≤12 months**		
	**value**	**95% CI**		**value**	**95% CI**
**Sensitivity**	0.9167	0.6461 to 0.9957	**Sensitivity**	1	0.7225 to 1.000
**Specificity**	0.9821	0.9055 to 0.9991	**Specificity**	0.75	0.5050 to 0.8982
**PPV**	0.9167	0.6461 to 0.9957	**PPV**	0.7143	0.4535 to 0.8828
**NPV**	0.9821	0.9055 to 0.9991	**NPV**	1	0.7575 to 1.000
**HR-ARM ≤24 months**			**CE ≤24 months**		
	**value**	**95% CI**		**value**	**95% CI**
**Sensitivity**	0.9333	0.7018 to 0.9966	**Sensitivity**	0.8462	0.5777 to 0.9727
**Specificity**	0.9577	0.8830 to 0.9885	**Specificity**	0.8095	0.6000 to 0.9233
**PPV**	0.8235	0.5897 to 0.9381	**PPV**	0.7333	0.4805 to 0.8910
**NPV**	0.9855	0.9224 to 0.9993	**NPV**	0.8947	0.6861 to 0.9813
**HR-ARM > 24 months**			**CE >24 months**		
	**value**	**95% CI**		**value**	**95% CI**
**Sensitivity**	1	0.05129 to 1.000	**Sensitivity**	0	0.000 to 0.9487
**Specificity**	0.7955	0.6550 to 0.8885	**Specificity**	0.8182	0.6148 to 0.9269
**PPV**	0.1	0.00512 to 0.4042	**PPV**	0	0.000 to 0.4899
**NPV**	1	0.9011 to 1.000	**NPV**	0.9474	0.7536 to 0.9973
